# Methyl 2-(5-bromo-2-methyl­naphtho[2,1-*b*]furan-1-yl)acetate

**DOI:** 10.1107/S1600536808015511

**Published:** 2008-05-30

**Authors:** Martyn Jevric, Dennis K. Taylor, Edward R. T. Tiekink

**Affiliations:** a Department of Chemistry, The University of Adelaide, 5005 South Australia, Australia; b Department of Wine and Horticulture, The University of Adelaide, Waite Campus, Glen Osmond 5064, South Australia, Australia; c Department of Chemistry, The University of Texas at San Antonio, One UTSA Circle, San Antonio, Texas 78249-0698, USA

## Abstract

The three fused six-, six- and five-membered rings in the title compound, C_16_H_13_BrO_3_, are coplanar, the CH_2_C(=O)OCH_3_ residue being twisted out of this plane [dihedral angle = −26.9 (4)°]. Centrosymmetric dimers are found in the crystal structure stabilized by C—H⋯O inter­actions involving the furan O atom.

## Related literature

For related literature, see: Chatterjea *et al.* (1979 ▶); Einhorn *et al.* (1983 ▶); Monte *et al.* (1996 ▶); Jevric *et al.* (2008 ▶).
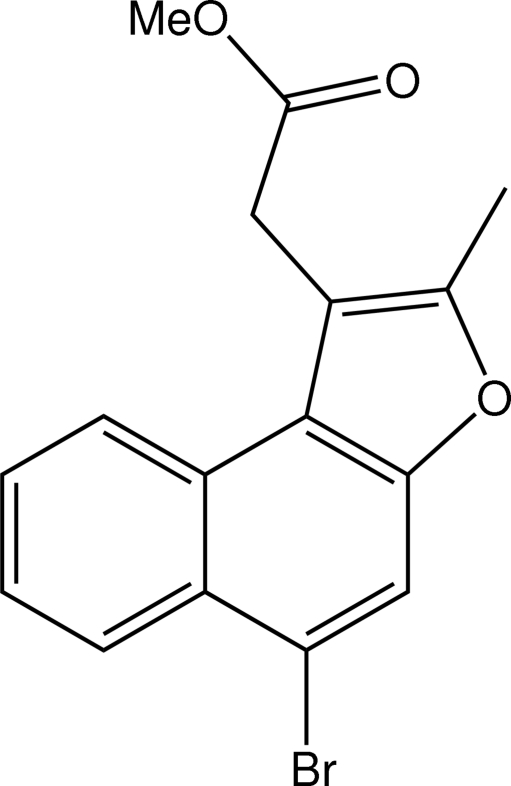


## Experimental

### 

#### Crystal data

C_16_H_13_BrO_3_
*M* *_r_* = 333.17
Monoclinic, 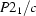

*a* = 17.050 (2) Å
*b* = 14.5064 (17) Å
*c* = 5.3660 (7) Å
β = 96.443 (3)°
*V* = 1318.8 (3) Å^3^
*Z* = 4
Mo *K*α radiation
μ = 3.12 mm^−1^
*T* = 223 (2) K
0.68 × 0.18 × 0.16 mm

#### Data collection

Bruker SMART CCD diffractometer
Absorption correction: multi-scan (*SADABS*; Bruker, 2000 ▶) *T*_min_ = 0.492, *T*_max_ = 1.000 (expected range = 0.299–0.607)
10676 measured reflections
3817 independent reflections
2967 reflections with *I* > 2σ(*I*)
*R*_int_ = 0.035

#### Refinement

*R*[*F* ^2^ > 2σ(*F* ^2^)] = 0.044
*wR*(*F* ^2^) = 0.136
*S* = 1.13
3817 reflections
183 parameters
H-atom parameters constrained
Δρ_max_ = 0.47 e Å^−3^
Δρ_min_ = −0.77 e Å^−3^

### 

Data collection: *SMART* (Bruker, 2000 ▶); cell refinement: *SAINT* (Bruker, 2000 ▶); data reduction: *SAINT*; program(s) used to solve structure: *SIR92* (Altomare *et al.*, 1994 ▶); program(s) used to refine structure: *SHELXL97* (Sheldrick, 2008 ▶); molecular graphics: *ORTEPII* (Johnson, 1976 ▶) and *DIAMOND* (Brandenburg, 2006 ▶); software used to prepare material for publication: *SHELXL97*.

## Supplementary Material

Crystal structure: contains datablocks global, I. DOI: 10.1107/S1600536808015511/su2059sup1.cif

Structure factors: contains datablocks I. DOI: 10.1107/S1600536808015511/su2059Isup2.hkl

Additional supplementary materials:  crystallographic information; 3D view; checkCIF report

## Figures and Tables

**Table 1:** Hydrogen-bond geometry (Å, °)

*D*—H⋯*A*	*D*—H	H⋯*A*	*D*⋯*A*	*D*—H⋯*A*
C4—H4⋯O3^i^	0.94	2.58	3.468 (4)	157

Symmetry code: (i) 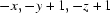.

**Fig. 1.**
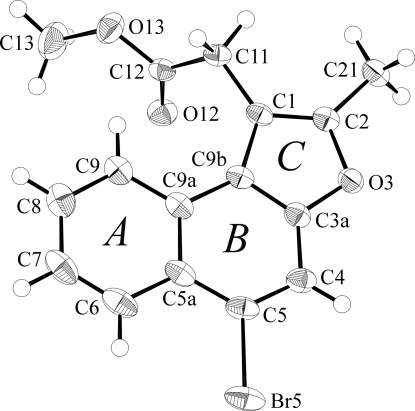
The molecular structure of compound (I) showing the atom-labelling scheme and displacement ellipsoids drawn at the 50% probability level.

**Fig. 2.**
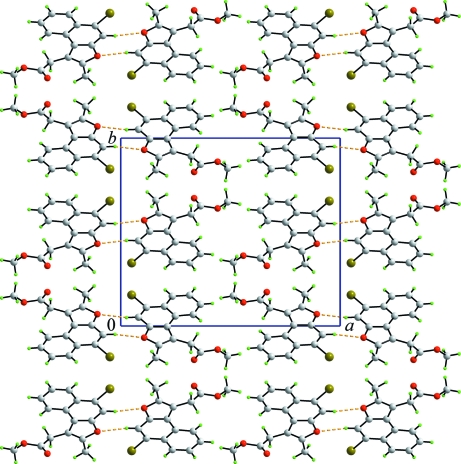
Crystal packing of compound (I) viewed in projection down the c axis, highlighting the stacking of the dimeric aggregates. Colour scheme olive (Br), red (O), grey (C), and green (H). The C—H···O contacts are shown as orange dashed lines.

**Fig. 3.**
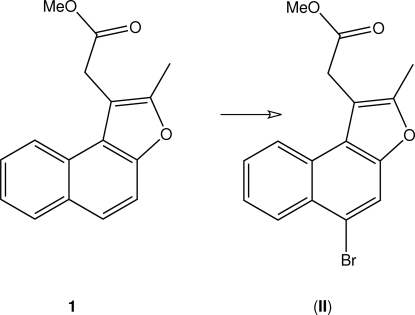
The formation of the title compound.

## supplementary crystallographic information

### Comment

Little has been done on observing aromatic electrophillic substitutions on polycyclic aromatic systems related to 1 shown in Fig. 3 (Chatterjea *et al.*, 1979). Previous work showed that substitution should proceed at position five in the ring system of 1 (Chatterjea *et al.*, 1979). Treatment of 1 with bromine in acetic acid according to a literature procedure (Einhorn *et al.*, 1983 & Monte *et al.*, 1996) gave the title compound (I) as the sole isolatable product. ^1^H NMR analysis of (I) showed five aromatic proton signals, two of which experienced two large *ortho* couplings and one a singlet (δ 7.81). This coupling pattern indicated that an aromatic electrophilic substitution had occurred on the ring adjoining the furan. However, although all signals were unobscured it was not possible to assign the *peri* proton as no detectable cross-peak in the ROESY spectrum was observed. X-ray crystallography showed that the position of the bromine substitution was in accordance with previous literature (Chatterjea *et al.*, 1979).

Compound (I), Fig. 1, is comprised of three fused rings; two six-membered rings (A & B) and one five-membered ring (C). The respective A/B, A/C & B/C dihedral angles between their least-squares planes are 1.88 (13), 4.16 (15) & 2.48 (14)°. The CH_2_C(=O)OCH_3_ residue is twisted out of the tricyclic system, as seen in the value of the C1/C11/C12/O12 torsion angle of -26.9 (4)°. The crystal packing features centrosymmetric dimers consolidated by C—H···O contacts involving the furan-O atom; Table 1 and Fig. 2.

The structure ofthe related compound 2-(2-methylnaphtho[2,1-*b*]furan-1-yl)acetic acid has been reported in the preceding paper (Jevric *et al.*, 2008).

### Experimental

Compound (I) was formed (Einhorn *et al.*, 1983 & Monte *et al.*, 1996) in 88% yield as a colourless solid recrystallized from n-heptane; m.p.: 391 - 393 K. *R*_f_ = 0.24 (12% acetone in hexane). IR (CH_2_Cl_2_, cm^-1^) 1741, 1618, 1577, 1521. ^1^H NMR (d_6_-benzene, 600 MHz) δ 2.00 (s, 3H), 3.21 (s, 3H), 3.41 (s, 2H), 7.27 (ddd, J = 7.0, 7.0, 1.2 Hz, 1H), 7.38 (ddd, J = 7.0, 7.0, 1.2 Hz, 1H), 7.81 (s, 1H), 8.36 (dd, J = 7.0, 1.2 Hz, 1H), 8.46 (dd, J = 7.0, 1.2 Hz, 1H) p.p.m.. ^13^C NMR (CDCl_3_, 50 MHz) δ 11.9, 31.4, 52.3, 109.5, 116.5, 118.1, 122.3, 123.1, 125.2, 126.9, 128.3, 128.4, 128.8, 150.7, 152.9, 171.3 p.p.m.. MS m/*z* (%): 332 (*M*+, 100), 273 (93), 259 (25), 194 (37), 165 (62). HRMS, C_16_H_13_BrO_3_: calcd, 332.0049; found 332.0062.

### Refinement

All H atoms were included in calculated positions and treated as riding atoms: C—H = 0.94 - 0.98 Å, and with *U*_iso_(H) = 1.5 or 1.2*U*_eq_(C).

### Crystal data

**Table:**

C_16_H_13_BrO_3_	*F*_000_ = 672
*M**_r_* = 333.17	*D*_x_ = 1.678 Mg m^−^^3^
Monoclinic, *P*2_1_/*c*	Mo *K*α radiation λ = 0.71069 Å
Hall symbol: -P 2ybc	Cell parameters from 2984 reflections
*a* = 17.050 (2) Å	θ = 2.4–29.7º
*b* = 14.5064 (17) Å	µ = 3.12 mm^−^^1^
*c* = 5.3660 (7) Å	*T* = 223 (2) K
β = 96.443 (3)º	Plate, pale-yellow
*V* = 1318.8 (3) Å^3^	0.68 × 0.18 × 0.16 mm
*Z* = 4	

### Data collection

**Table:**

Bruker SMART CCD diffractometer	3817 independent reflections
Radiation source: fine-focus sealed tube	2967 reflections with *I* > 2σ(*I*)
Monochromator: graphite	*R*_int_ = 0.035
*T* = 223(2) K	θ_max_ = 30.1º
ω scans	θ_min_ = 1.2º
Absorption correction: multi-scan(SADABS; Bruker, 2000)	*h* = −23→23
*T*_min_ = 0.492, *T*_max_ = 1.0	*k* = −14→20
10676 measured reflections	*l* = −7→7

### Refinement

**Table:**

Refinement on *F*^2^	Secondary atom site location: difference Fourier map
Least-squares matrix: full	Hydrogen site location: inferred from neighbouring sites
*R*[*F*^2^ > 2σ(*F*^2^)] = 0.044	H-atom parameters constrained
*wR*(*F*^2^) = 0.136	*w* = 1/[σ^2^(*F*_o_^2^) + (0.0586*P*)^2^ + 0.71*P*] where *P* = (*F*_o_^2^ + 2*F*_c_^2^)/3
*S* = 1.13	(Δ/σ)_max_ < 0.001
3817 reflections	Δρ_max_ = 0.47 e Å^−^^3^
183 parameters	Δρ_min_ = −0.77 e Å^−^^3^
Primary atom site location: structure-invariant direct methods	Extinction correction: none

### Special details

**Table:**

Geometry. All e.s.d.'s (except the e.s.d. in the dihedral angle between two l.s. planes) are estimated using the full covariance matrix. The cell e.s.d.'s are taken into account individually in the estimation of e.s.d.'s in distances, angles and torsion angles; correlations between e.s.d.'s in cell parameters are only used when they are defined by crystal symmetry. An approximate (isotropic) treatment of cell e.s.d.'s is used for estimating e.s.d.'s involving l.s. planes.
Refinement. Refinement of *F*^2^ against ALL reflections. The weighted *R*-factor *wR* and goodness of fit *S* are based on *F*^2^, conventional *R*-factors *R* are based on *F*, with *F* set to zero for negative *F*^2^. The threshold expression of *F*^2^ > σ(*F*^2^) is used only for calculating *R*-factors(gt) *etc*. and is not relevant to the choice of reflections for refinement. *R*-factors based on *F*^2^ are statistically about twice as large as those based on *F*, and *R*- factors based on ALL data will be even larger.

### Fractional atomic coordinates and isotropic or equivalent isotropic displacement parameters (Å^2^)

**Table:**

	*x*	*y*	*z*	*U*_iso_*/*U*_eq_	
Br5	0.04963 (2)	0.33514 (2)	−0.16830 (6)	0.04284 (14)	
O3	0.10671 (11)	0.56263 (13)	0.5779 (4)	0.0285 (4)	
O12	0.33445 (13)	0.68019 (14)	0.3251 (4)	0.0321 (4)	
O13	0.44151 (12)	0.62588 (17)	0.5516 (4)	0.0379 (5)	
C1	0.23909 (16)	0.56919 (18)	0.6116 (5)	0.0242 (5)	
C2	0.17380 (16)	0.60179 (18)	0.7005 (5)	0.0258 (5)	
C3A	0.13162 (16)	0.50429 (18)	0.4058 (5)	0.0262 (5)	
C4	0.08136 (18)	0.45375 (19)	0.2380 (5)	0.0308 (6)	
H4	0.0263	0.4554	0.2388	0.037*	
C5	0.11641 (18)	0.40163 (19)	0.0720 (5)	0.0307 (6)	
C5A	0.19934 (18)	0.39606 (18)	0.0699 (5)	0.0293 (6)	
C6	0.2347 (2)	0.34180 (19)	−0.1028 (6)	0.0384 (7)	
H6	0.2027	0.3084	−0.2251	0.046*	
C7	0.3142 (2)	0.3368 (2)	−0.0963 (6)	0.0422 (8)	
H7	0.3368	0.3005	−0.2146	0.051*	
C8	0.3627 (2)	0.3850 (2)	0.0844 (6)	0.0371 (7)	
H8	0.4178	0.3802	0.0899	0.044*	
C9	0.33055 (18)	0.43933 (19)	0.2536 (5)	0.0306 (6)	
H9	0.3639	0.4719	0.3744	0.037*	
C9A	0.24847 (17)	0.44736 (18)	0.2501 (5)	0.0268 (5)	
C9B	0.21225 (16)	0.50423 (17)	0.4179 (5)	0.0242 (5)	
C11	0.32098 (16)	0.59630 (19)	0.7023 (5)	0.0259 (5)	
H11A	0.3502	0.5416	0.7670	0.031*	
H11B	0.3199	0.6399	0.8413	0.031*	
C12	0.36339 (16)	0.63946 (18)	0.5035 (5)	0.0258 (5)	
C13	0.4870 (2)	0.6604 (3)	0.3638 (8)	0.0546 (10)	
H13A	0.4710	0.6295	0.2058	0.082*	
H13B	0.5425	0.6489	0.4141	0.082*	
H13C	0.4782	0.7262	0.3438	0.082*	
C21	0.16031 (19)	0.66841 (19)	0.8973 (6)	0.0315 (6)	
H21A	0.1419	0.6362	1.0380	0.047*	
H21B	0.1209	0.7129	0.8313	0.047*	
H21C	0.2093	0.7001	0.9526	0.047*	

### Atomic displacement parameters (Å^2^)

**Table:**

	*U*^11^	*U*^22^	*U*^33^	*U*^12^	*U*^13^	*U*^23^
Br5	0.0624 (3)	0.0341 (2)	0.02918 (18)	−0.01456 (14)	−0.00759 (14)	−0.00306 (11)
O3	0.0299 (10)	0.0309 (10)	0.0251 (9)	−0.0050 (8)	0.0054 (7)	−0.0038 (8)
O12	0.0351 (11)	0.0337 (11)	0.0268 (10)	0.0006 (8)	0.0003 (8)	0.0070 (8)
O13	0.0265 (10)	0.0498 (13)	0.0375 (12)	0.0037 (9)	0.0032 (9)	0.0119 (10)
C1	0.0328 (14)	0.0211 (11)	0.0183 (11)	−0.0022 (10)	0.0018 (9)	0.0015 (9)
C2	0.0310 (13)	0.0232 (12)	0.0231 (12)	−0.0028 (10)	0.0025 (10)	0.0012 (9)
C3A	0.0322 (14)	0.0246 (12)	0.0221 (11)	−0.0042 (10)	0.0047 (10)	0.0012 (10)
C4	0.0345 (15)	0.0304 (14)	0.0267 (13)	−0.0095 (11)	0.0005 (11)	0.0013 (11)
C5	0.0427 (16)	0.0240 (12)	0.0238 (12)	−0.0093 (11)	−0.0036 (11)	0.0019 (10)
C5A	0.0463 (16)	0.0208 (12)	0.0206 (12)	−0.0010 (11)	0.0035 (11)	0.0027 (9)
C6	0.064 (2)	0.0249 (14)	0.0262 (14)	0.0001 (13)	0.0036 (13)	−0.0017 (11)
C7	0.069 (2)	0.0293 (15)	0.0304 (15)	0.0110 (15)	0.0131 (15)	−0.0013 (12)
C8	0.0463 (17)	0.0279 (14)	0.0382 (16)	0.0105 (13)	0.0101 (13)	0.0048 (12)
C9	0.0400 (15)	0.0239 (13)	0.0283 (13)	0.0031 (11)	0.0058 (11)	0.0036 (10)
C9A	0.0383 (15)	0.0198 (12)	0.0227 (12)	0.0002 (10)	0.0052 (10)	0.0030 (9)
C9B	0.0333 (14)	0.0191 (11)	0.0198 (11)	−0.0032 (10)	0.0010 (10)	0.0022 (9)
C11	0.0298 (13)	0.0262 (13)	0.0207 (11)	−0.0016 (10)	−0.0020 (10)	0.0021 (9)
C12	0.0294 (13)	0.0232 (12)	0.0239 (12)	0.0003 (10)	−0.0009 (10)	−0.0032 (9)
C13	0.0363 (18)	0.079 (3)	0.051 (2)	−0.0019 (17)	0.0130 (16)	0.0162 (19)
C21	0.0381 (15)	0.0305 (14)	0.0265 (13)	−0.0039 (12)	0.0068 (11)	−0.0034 (11)

### Geometric parameters (Å, °)

**Table:**

Br5—C5	1.887 (3)	C6—H6	0.9400
O3—C3A	1.355 (3)	C7—C8	1.390 (5)
O3—C2	1.377 (3)	C7—H7	0.9400
O12—C12	1.184 (3)	C8—C9	1.362 (4)
O13—C12	1.343 (3)	C8—H8	0.9400
O13—C13	1.429 (4)	C9—C9A	1.402 (4)
C1—C2	1.345 (4)	C9—H9	0.9400
C1—C9B	1.439 (3)	C9A—C9B	1.413 (4)
C1—C11	1.479 (4)	C11—C12	1.492 (4)
C2—C21	1.468 (4)	C11—H11A	0.9800
C3A—C9B	1.369 (4)	C11—H11B	0.9800
C3A—C4	1.382 (4)	C13—H13A	0.9700
C4—C5	1.357 (4)	C13—H13B	0.9700
C4—H4	0.9400	C13—H13C	0.9700
C5—C5A	1.417 (4)	C21—H21A	0.9700
C5A—C6	1.403 (4)	C21—H21B	0.9700
C5A—C9A	1.417 (4)	C21—H21C	0.9700
C6—C7	1.354 (6)		
			
C3A—O3—C2	106.0 (2)	C8—C9—H9	119.5
C12—O13—C13	114.7 (2)	C9A—C9—H9	119.5
C2—C1—C9B	106.1 (2)	C9—C9A—C9B	123.1 (3)
C2—C1—C11	125.3 (2)	C9—C9A—C5A	118.6 (3)
C9B—C1—C11	128.6 (2)	C9B—C9A—C5A	118.3 (3)
C1—C2—O3	111.2 (2)	C3A—C9B—C9A	118.6 (2)
C1—C2—C21	133.6 (3)	C3A—C9B—C1	105.7 (2)
O3—C2—C21	115.2 (2)	C9A—C9B—C1	135.7 (3)
O3—C3A—C9B	111.0 (2)	C1—C11—C12	113.1 (2)
O3—C3A—C4	123.8 (3)	C1—C11—H11A	109.0
C9B—C3A—C4	125.2 (3)	C12—C11—H11A	109.0
C5—C4—C3A	115.9 (3)	C1—C11—H11B	109.0
C5—C4—H4	122.1	C12—C11—H11B	109.0
C3A—C4—H4	122.1	H11A—C11—H11B	107.8
C4—C5—C5A	123.4 (3)	O12—C12—O13	122.9 (3)
C4—C5—Br5	117.2 (2)	O12—C12—C11	126.6 (3)
C5A—C5—Br5	119.4 (2)	O13—C12—C11	110.6 (2)
C6—C5A—C9A	118.7 (3)	O13—C13—H13A	109.5
C6—C5A—C5	122.7 (3)	O13—C13—H13B	109.5
C9A—C5A—C5	118.6 (3)	H13A—C13—H13B	109.5
C7—C6—C5A	121.1 (3)	O13—C13—H13C	109.5
C7—C6—H6	119.5	H13A—C13—H13C	109.5
C5A—C6—H6	119.5	H13B—C13—H13C	109.5
C6—C7—C8	120.4 (3)	C2—C21—H21A	109.5
C6—C7—H7	119.8	C2—C21—H21B	109.5
C8—C7—H7	119.8	H21A—C21—H21B	109.5
C9—C8—C7	120.2 (3)	C2—C21—H21C	109.5
C9—C8—H8	119.9	H21A—C21—H21C	109.5
C7—C8—H8	119.9	H21B—C21—H21C	109.5
C8—C9—C9A	121.0 (3)		

### Hydrogen-bond geometry (Å, °)

**Table:**

*D*—H···*A*	*D*—H	H···*A*	*D*···*A*	*D*—H···*A*
C4—H4···O3^i^	0.94	2.58	3.468 (4)	157

Symmetry codes: (i) −*x*, −*y*+1, −*z*+1.
